# Role of Macrophage Colony-Stimulating Factor Receptor on the Proliferation and Survival of Microglia Following Systemic Nerve and Cuprizone-Induced Injuries

**DOI:** 10.3389/fimmu.2020.00047

**Published:** 2020-01-29

**Authors:** Vincent Pons, Nataly Laflamme, Paul Préfontaine, Serge Rivest

**Affiliations:** Neuroscience Laboratory, Department of Molecular Medicine, Faculty of Medicine, CHU de Québec Research Center, Laval University, Québec City, QC, Canada

**Keywords:** microglia, proliferation, brain injuries, demyelination, monocytes, CSF1R

## Abstract

Microglia are the innate immune cells of the CNS and their proliferation, activation, and survival have previously been shown to be highly dependent on macrophage colony-stimulating factor receptor (CSF1R). Here we investigated the impact of the receptor in such processes using two different models of nerve injuries, namely hypoglossal axotomy and cuprizone-induced demyelination. Both models are associated with a robust microgliosis. The role of CSF1R was investigated using the gene deletion Cre/Lox system, which allows the conditional knock-out following tamoxifen administration. We found that after 5 weeks of cuprizone diet that CSF1R suppression caused a significant impairment of microglia function. A reduced microgliosis was detected in the corpus collosum of CSF1R knock-out mice compared to controls. In contrast to cuprizone model, the overall number of Iba1 cells was unchanged at all the times evaluated following hypoglossal axotomy in WT and cKO conditions. After nerve lesion, a tremendous proliferation was noticed in the ipsilateral hypoglossal nucleus to a similar level in both knock-out and wild-type groups. We also observed infiltration of bone-marrow derived cells specifically in CSF1R-deficient mice, these cells tend to compensate the CSF1R signaling pathway suppression in resident microglia. Taking together our results suggest a different role of CSF1R in microglia depending on the model. In the pathologic context of cuprizone-induced demyelination CSF1R signaling pathway is essential to trigger proliferation and survival of microglia, while this is not the case in a model of systemic nerve injury. M-CSF/CSF1R is consequently not the unique system involved in microgliosis following nerve damages.

## Introduction

Macrophage colony-stimulating factor receptor (CSF1R) is a receptor of the tyrosine kinase family. It is broadly expressed in the organism by monocytes, resident macrophages, osteoclasts, Paneth cells, dendritic cells, and in the brain microglia. Two ligands bind CSF1R, macrophage colony-stimulating factor (mCSF) and Interleukine-34 (IL-34), which have complementary roles on the proliferation of innate immune cells, especially on monocyte and macrophage populations (1). In the brain, it acts on the phagocytosis, survival, and proliferation of microglia (2). Microglia are the resident immune cells of the brain. These mononuclear phagocytes arise from hematopoietic progenitors in the yolk sac during embryogenesis and are generated at the postnatal stage. These immune cells are implicated in brain homeostasis, and can detect any inflammatory and damage sites (3).

Microglia and CSF1R signaling pathway are involved in different neuroprotective roles, such as clearing myelin debris and toxic proteins from the cerebral environment. In this regard, modulation of the receptor is thought to be a novel therapeutic avenue for diseases, such as Alzheimer's disease (AD), multiple sclerosis (MS), and brain tumors (2, 4–7). To better understand how CSF1R drives proliferation of microglial cells, we have deleted this receptor specifically in CX3CR1-postive cells in different nerve damage and pathologic models. Hypoglossal axotomy model provides a sterile proliferative system to better understand the cellular and molecular events associated with microglia proliferation, without interfering with the environment found from pathological models (8). After the systemic nerve section, a tremendous microglial proliferation takes place in the ipsilateral hypoglossal nucleus. The cuprizone model allows to study cellular and molecular mechanisms involved in the demyelination/remyelination processes, while excluding the autoimmune component. Here also there is a robust microgliosis in brain regions where myelin and oligodendrocytes are affected by the copper-chelating toxin (9).

Here we have investigated the role of CSF1R on the proliferation of microglia using the gene deletion Cre/Lox system, which allows the knock-out following tamoxifen administration. We used two different models causing microglial cell proliferation to determine whether CSF1R is crucial for both sterile and toxin cues. Our results show that CSF1R is essential for microglia proliferation in cuprizone-fed mice indicating that this signaling pathway in such a mouse model of progressing MS is vital for microglial survival and proliferation. Surprisingly deletion of this receptor has no impact on such cellular functions following hypoglossal nerve injury. Moreover, a compensatory mechanism seems to take place for the loss of CSF1R with overexpression of TREM2 in microglia.

## Materials and Methods

### Animals Surgery

Animals were injected with tamoxifen following several time-course (**Figure 2**). Males, 2 to 4-month-old mice were anesthetized using isofluorane (Baxter Corporation, Ontario, Canada) 3–4% and oxygen 0.8–1.5 l/min, then they were shaved. To mitigate the pain, we used Maxilene®4, lidocaine cream 4% (Ferndale Laboratories, Inc., MI 48220 USA), applied on the neck 5 min prior the surgery. After the pre-surgery preparation, isofluorane is set to 1.5–2% and oxygen flux was adjusted to 1.5–2 l/min. Animals were placed in the supine position, and the right hypoglossal nerve was transected with scissors. Mice were kept alive 1 week after surgery.

### Cuprizone Diet

0.2% wt/wt cuprizone (bis-cyclohexylidene hydrazide; Sigma-Aldrich) was mixed with regular ground irradiated chow and fed to experimental animals for 5 weeks. The chow was changed every 2 days and food intake was monitored throughout the protocols. Control animals were fed with regular irradiated ground chow and manipulated as often as cuprizone-fed mice. After terminating the 5 weeks of cuprizone diet mice were euthanized.

### Conditional CSF1R KO Mice

B6.Cg-Csf1r *tm1jwp*/J mice (JaxMice; stock number 02212) were crossed with the B6.129-Cx3cr1tm2.1 (CreER)Jung/Orl mice (EMMA mouse respiratory; EM:06350). The resulting mouse has a tamoxifen-inducible CRE activity specifically in microglial cells, leading to a non-functional CRF1R protein.

### Tamoxifen Preparation and Administration

Tamoxifen was dissolved in corn oil and Ethanol 100% for 1 h at 37 degrees, vortexed every 15 min. We used ~75 mg tamoxifen/Kg body weight and 100 μl tamoxifen/corn oil solution was administered via intraperitoneal injection for 4 consecutive days. Tamoxifen was injected 4 days before cuprizone diet, and 7, 13, or 21 days before nerve transection. For the hypoglossal nerve lesion, protocols are named, respectively, Short Protocol (SP), Principal protocol (P1), and Long Protocol (LP).

### Chimeric Mice

Experimental animals received a total of 80 mg/kg of Busulfan administered i.p. every 12 h for 4 days, followed by 2 days of single i.p. injection of 100 mg/kg cyclophosphamide. After a 24-h rest, 3 × 10^7^ bone marrow cells isolated from the tibia and femur of donor mice were injected into the tail vein of target animals. C57BL/6-Tg (CAG-EGFP) 1O sb/J (JaxMice stock number 003291) mice were used as donors. For details on this procedure, please refer to Laflamme et al. (10).

### Sacrifices

All mice were deeply anesthetized with ketamine/xylazine and sacrificed via intracardiac perfusion with 0.9% saline followed by 4% PFA pH 7.4 or pH 9. The brains were then retrieved, post-fixed 10–24 h in 4% PFA pH 7.4 and transferred in 4% PFA pH 7.4 + 20% sucrose for a minimum of 15 h. Brains were sliced in coronal sections of 20-μm thickness with a freezing microtome (Leica Microsystems), serially collected in anti-freeze solution and kept at −20°C until usage.

### Immunohistochemical Staining

Brain sections were washed (4 × 5 min) in KPBS. An antigen retrieval step was performed to stain for CSF1R. More specifically, sections were boiled 10 min in sodium citrate 10 mM pH 6 just before the blocking step and then blocked in KPBS containing 1% BSA, and 1% Triton X-100. The tissues were then incubated overnight at 4°C with the primary antibody anti-Iba-1 (rabbit, 1:1,000; WAKO Chemical 019-19741), or with the primary antibody anti-CSF1R (sheep, 1:500; R&D System AF3818), or with the primary antibody Tmem119 (rabbit, 1:1,000; ABCAM ab209064). After washing the sections in KPBS (4 × 5 min), tissues were incubated in the appropriate secondary antibody (biotinylated goat anti-rabbit IgG; 1:1,500, Vector Laboratories. biotinylated rabbit anti-sheep IgG; 1:1,500, Vector Laboratories) for 2 h at room temperature. Following further washes in KPBS and 1 h-long incubation in avidin-biotin peroxidase complex (ABC; Vector Laboratories) the sections were then incubated in 3,3′-diaminobenzidine tetrahydrochloride (DAB; Sigma) to reveal the staining. The sections were mounted onto Micro Slides Superfrost® plus glass slides, dehydrated and then coverslipped with DPX mounting media.

### Immunofluorescent Staining

Brain sections were washed (4 × 5 min) in KPBS then blocked in KPBS containing 1% BSA, and 1% Triton X-100. The tissues were then incubated overnight at 4°C with the primary antibody anti-Iba-1 (rabbit, 1:1,000; WAKO Chemical 019-19741). After washing the section in KPBS (4 × 5 min), the tissue was incubated in the appropriate secondary antibody (IgG anti-rabbit Alexa 546; Invitrogen A11010) for 2 h at room temperature. Following further washes in KPBS and incubation with DAPI to identify the nuclei, the sections were mounted onto Micro Slides Superfrost® Plus glass slides and coverslipped with Fluoromount-G (Electron Microscopy Sciences).

### Western Blot

Brain protein lysates were extracted as previously described (11). Proteins were then loaded in 8–16% agarose precast gels (Biorad) and electroblotted onto 0.45 μm Immobilon PVDF membranes. Membranes were immunoblotted with primary antibodies anti-Iba-1 (rabbit, 1:1,000; WAKO Chemical 019-19741), followed by the appropriate horseradish peroxidase (HRP)-conjugated secondary antibodies and revealed by Clarity western (ECL) substrate (biorad). Quantification was done by determining integrative density of the bands using Thermo Scientific Pierce my Image Analysis Software v2.0. Optical values were normalized over actin.

### Quantitative Real-Time PCR

Tissues were homogenized in Qiazol buffer (Qiagen, Germantown, MD, USA) and total RNA was extracted using the miRNeasy micro kit on-column DNase (Qiagen, Hilden, DE) treatment following the manufacturer's instructions. Quantity of total RNA was measured using a NanoDrop ND-1000 Spectrophotometer (NanoDrop Technologies, Wilmington, DE, USA) and total RNA quality was assayed on an Agilent BioAnalyzer 2100 (Agilent Technologies, Santa Clara, CA, USA).

First-strand cDNA synthesis was accomplished using 4 μg of isolated RNA in a reaction containing 200 U of Superscript IV Rnase H-RT (Invitrogen Life Technologies, Burlington, ON, CA), 300 ng of oligo-dT_18_, 50 ng of random hexamers, 50 mM Tris-HCl pH 8.3, 75 mM KCl, 3 mM MgCl_2_, 500 μM deoxynucleotides triphosphate, 5 mM dithiothreitol, and 40 U of Protector RNase inhibitor (Roche Diagnostics, Indianapolis, IN, USA) in a final volume of 50 μl. Reaction was incubated at 25°C for 10 min, then at 50°C for 20 min, inactivated at 80°C for 10 min. PCR purification kit (Qiagen, Hilden, DE) was used to purify cDNA.

Oligoprimer pairs were performed by IDT (Integrated DNA Technology, Coralville, IA, USA) (Table 1). A quantity corresponding to 20 ng of total RNA was used to perform fluorescent-based Realtime PCR quantification using the LightCycler 480 (Roche Diagnostics, Mannheim, DE). Reagent LightCycler 480 SYBRGreen I Master (Roche Diagnostics, Indianapolis, IN, USA) was used as described by the manufacturer with 2% DMSO. The conditions for PCR reactions were: 45 cycles, denaturation at 95°C for 10 s, annealing at 60°C for 10 s, elongation at 72°C for 14 s and then 74°C for 5 s (reading). A melting curve was performed to assess non-specific signal. Relative quantity was calculated using second derivative method and by applying the delta Ct (12). Normalization was performed using the reference gene shown to be genes having stable expression levels from embryonic life through adulthood in various tissues (13) hypoxanthine guanine phosphoribosyl transferase 1 (HPRT1) and glyceraldehyde-3-phosphate dehydrogenase (GAPDH). Quantitative Real-Time PCR measurements were performed by the CHU de Québec Research Center *(CHUL)* Gene Expression Platform, Quebec, Canada and were compliant with MIQE guidelines.

**Table 1:** Sequence primers and gene description.

**Gene symbol**	**Description**	**GenBank**	**Size (pb)**	**Primer sequence 5^**′**^ → 3^**′**^** ** S/AS**
Tmem119	Mus musculus transmembrane protein 19 (Tmem19)	NM_133683	121	ACTGCTTCCTGGATGTGTTTGTCTC/ CCCAGGTTGTTATTAGCCGAGGT
TREM2	Mus musculus triggering receptor expressed on myeloid cells 2 (Trem2), 2 transcripts	NM_001272078	160	TGGTGTCGGCAGCTGGGTGAG/ CGGCTTGGAGGTTCTTCAGAGT
Hprt1	Mus musculus hypoxanthine guanine phosphoribosyl transferase 1	NM_013556	106	CAGGACTGAAAGACTTGCTCGAGAT/ CAGCAGGTCAGCAAAGAACTTATAGC
GAPDH	Mus musculus glyceraldehyde-3-phosphate dehydrogenase	NM_008084	194	ggctgcccagaacatcatccct/ atgcctgcttcaccaccttcttg
ADNg	Mus musculus chromosome 3 genomic contig, strain C57BL/6J (HSD3B1 intron)	NT_039239	209	CACCCCTTAAGAGACCCATGTT/ CCCTGCAGAGACCTTAGAAAAC

### Image Acquisition and Analyses

Image acquisition of Fluorescent staining images was performed using a Zeiss LSM800 confocal microscope supported by the Zen software (2.3 system) using the 10×, 20×, and 40× lenses. Confocal images were then processed using Fiji (ImageJ Version 2.0.0-rc-43/1.51n). For analyses and brightfield image acquisition of staining, Iba-1 and CSF1R, 8-bit grayscale TIFF images of the regions of interest were taken in a single sitting for whole protocols with a Qimaging camera (Qcapture program, version 2.9.10), attached to Nikon microscope (C-80) with the same gain/exposure settings for every image. To evaluate the level of Iba-1^+^ immune response in the regions of interest (corpus callosum and hypoglossal nucleus), the images were imported into ImageJ (1.37) and the percentage of area occupied by the staining was measured using the threshold parameter. Cells count was assed manually using ImageJ (1.37). Analysis was performed in double blinded to avoid bias of analysis, each microglial cell body in hypoglossal nucleus were counted. Fluorescent staining of images was performed using a Zeiss LSM800 confocal microscope supported by the Zen software (2.3 system). Confocal images were then processed using Fiji (ImageJ Version 2.0.0-rc-43/1.51n).

### Statistical Analyses and Figure Preparation

Data are presented as mean ± standard error of the mean (SEM). Statistical analyses were carried with the Prism software (version 8.0, GraphPad Software Inc.). Values were considered statistically significant if *p* < 0.05. All panels were assembled using Adobe Photoshop CC 2018 (version 19.1.0) and Adobe Illustrator CC 2018 (version 23.0.1).

## Results

### Mouse Model of CSF1R Deletion Specifically in Microglia

**Figure 1:**
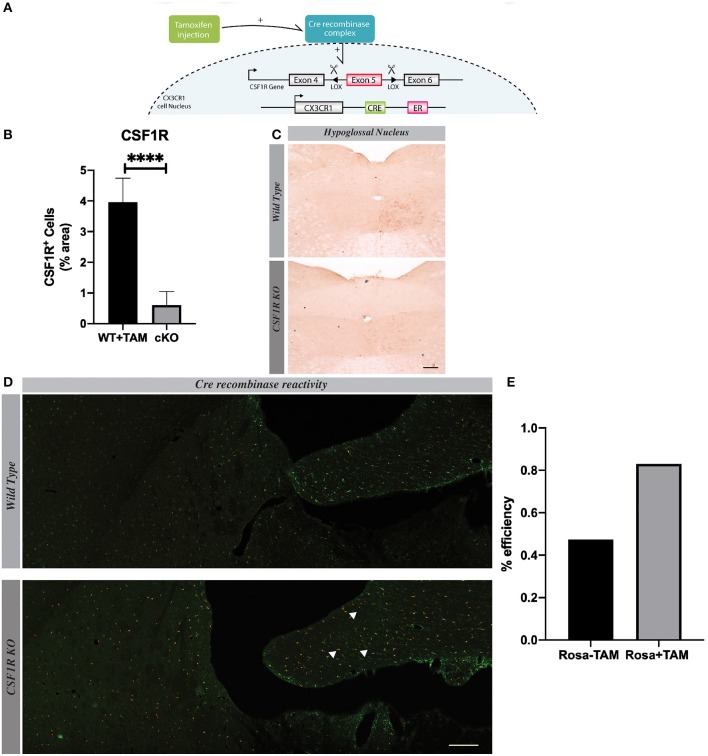
CSF1R is deleted specifically in microglia. **(A)** We Show the genetic construction of CSF1R Ko mice. **(B)** Quantification presented as percentage of area occupied by staining, measured in the hypoglossal nucleus. Values are expressed as means ± SEM. Statistical analyses were performed using *t*-test ^****^p < 0.001 significantly different from cKO group. **(C)** Representative images of CSF1R staining in hypoglossal nucleus 7 days after lesion. **(D)** Confocal images showing co-localization of RedTomato positive cells (red) with Iba1 immunoreactive cells (green). **(E)** Quantification of RedTomato staining. Mice were injected with tamoxifen. White arrows point-out some examples of co-localization. *n* = 7 mice. Scale bar 200 μm.

To delete CSF1R in microglia, we crossed CSF1R^fl/fl^ mice with the CX3CR1-Cre^ERT2^ mice and exposed them to i.p. tamoxifen (TAM) injections as previously reported by us (6). After injection, Cre complex goes to the nucleus and interacts with the Lox site, which leads to excision of CSF1R gene Exon 5 (Figure 1A). In order to determine when the knock-out affect a maximum of cells, we have performed three different time-courses (Supplementary Figure 1A). The surgery made 13 days after the last tamoxifen injection provided the best results. Quantification of CSF1R in the hypoglossal nucleus shows a strong effect of the knock-out since the CSF1R expression was dramatically decreased (Figures 1B,C). To further test the relevance of our model, we used CSF1R-loxP-CX3CR1-cre/ERT2,Rosa^tm14^ mice. Mice express robust tdTomato fluorescence following Cre-mediated recombination and a large amount of CX3CR1-positive cells in the brain are affected by the knock-out (Figure 1D). Quantification shows that 82.9% of Iba-1^+^ cells are Rosa^tm14+^ (Figure 1E). These data indicate that our model is reliable, and strongly efficient to delete CSFR1 selectively in microglia.

### Microglial Proliferation in Hypoglossal Nucleus Is Maximal 7 Days After the Lesion

**Figure 2:**
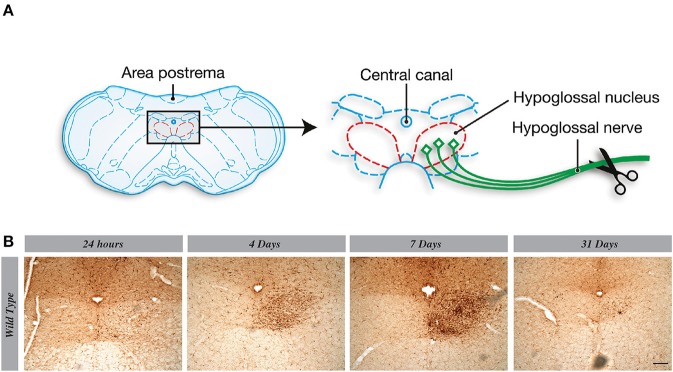
Microglial proliferation in hypoglossal nucleus is maximal 7 days after the lesion. **(A)** Scheme of hypoglossal nerve transection surgery. **(B)** A progressive increase of Iba1 staining is observed from 24 h to 7 days post axotomy, which declined at 31 days. *n* = 3 mice. Scale bar: 100 μm.

Hypoglossal nerve lesion causes a robust proliferation of microglia in the ipsilateral side of the nucleus, especially at time 7 days post injury (Figures 2A,B). Although few new cells are detected 24 h after the lesion, these are quite numerous at 4 and 7 days in the ipsilateral side of hypoglossal nerve-injured mice. After this time point, the number of new Iba1 positive cells slowly decreased to a basal level at day 31 after lesion (Figure 2B). Considering that the peak proliferating level is 7 days post-surgery, we selected this time point to determine the potential role of CSF1R in this mechanism.

### Knocking-Out CSF1R Selectively in Microglia Does Not Affect Cell Proliferation

**Figure 3:**
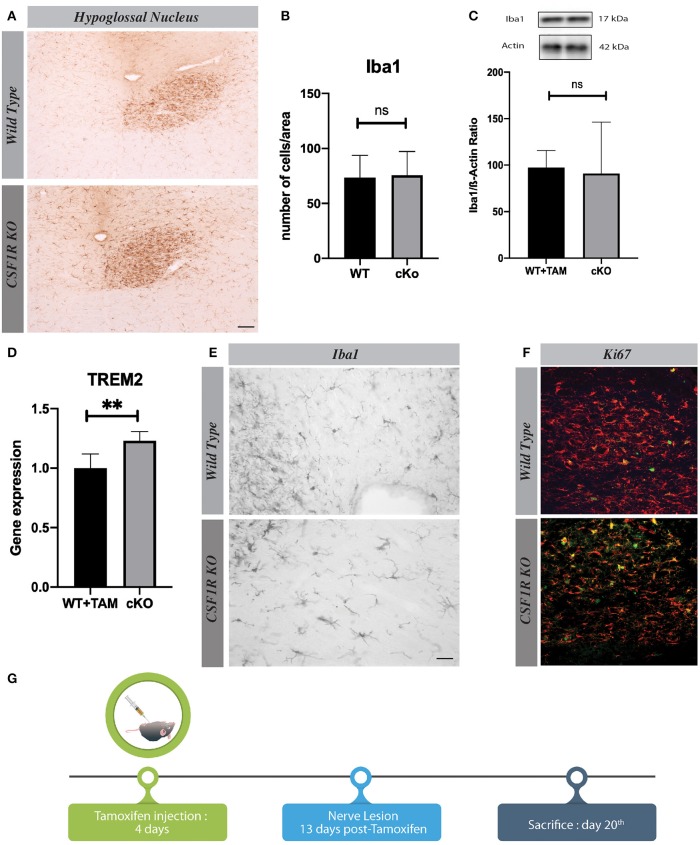
Knocking-out CSF1R selectively in microglia does not impair microglial cell proliferation. **(A)** Representative images of Iba1 staining in hypoglossal nucleus, using different timelines, and **(B)** their quantification presented as percentage of area occupied by Iba1 staining measured in hypoglossal nucleus. **(C)** Level of Iba1 in cerebellum by Western blot. **(D)** Quantification of TREM2 gene expression in cerebellum by RT-Q-PCR. **(E)** Representative images of microglial morphology in cKO and WT mice. **(F)** Representative images of Iba1 (red) and Ki67 (green) staining in hypoglossal nucleus area. **(G)** Time course used for analyses. Values are expressed as means ± SEM. Statistical analyses were performed using *t*-test, *p* < 0.005. Scale bar 100 μm. *n* = 3–5 mice. **p < 0.01.

As previously described, a marked proliferation of microglia takes place in the ipsilateral hypoglossal nucleus 7 days after the systemic nerve injury. Following our protocol, CSF1R-loxP-CX3CR1-cre/ERT2 mice had surgery 13 days after last tamoxifen injection to allow the complete knock-out in microglia (Figure 3G). Taking into consideration that CSF1R signaling pathway is known to be essential for microglia proliferation in pathological conditions (14, 15), our data are unexpected. Indeed, CSFR1 deletion did not affect microgliosis and a strong proliferation of Iba-1^+^ cells was observed in hypoglossal nucleus of CSF1R knock-out group (Figure 3A). There were actually no significant differences between WT and cKO groups (Figures 3B,F) and at all the time courses tested (Supplementary Figure 1). To further validate this surprising result, we determined the Iba-1 protein levels by Western blot and found a similar amount of Iba-1 levels in the brain of both groups of mice (Figure 3C). These results are quite interesting because they indicate that knocking-out CSF1R in microglial cells in a non-pathologic context does not impair their proliferation (Figure 3F). These findings put in light on the subjective importance of CSF1R signaling pathway depending on a pathologic or non-pathologic situation. We have also noticed that the morphology of microglia in the CSF1R cKO mice seemed different from those of control mice (Figure 3E). They have more ramifications and their cell bodies are thicker in CSF1R-deficient mice. This phenotype match with the shape observed in TREM2 overexpression cells (16) (Figure 3D) and this results could be the clue of a compensating mechanism overcoming the CSF1R deletion.

### CSF1R Depletion Induces Infiltration of Peripheral Cells

**Figure 4:**
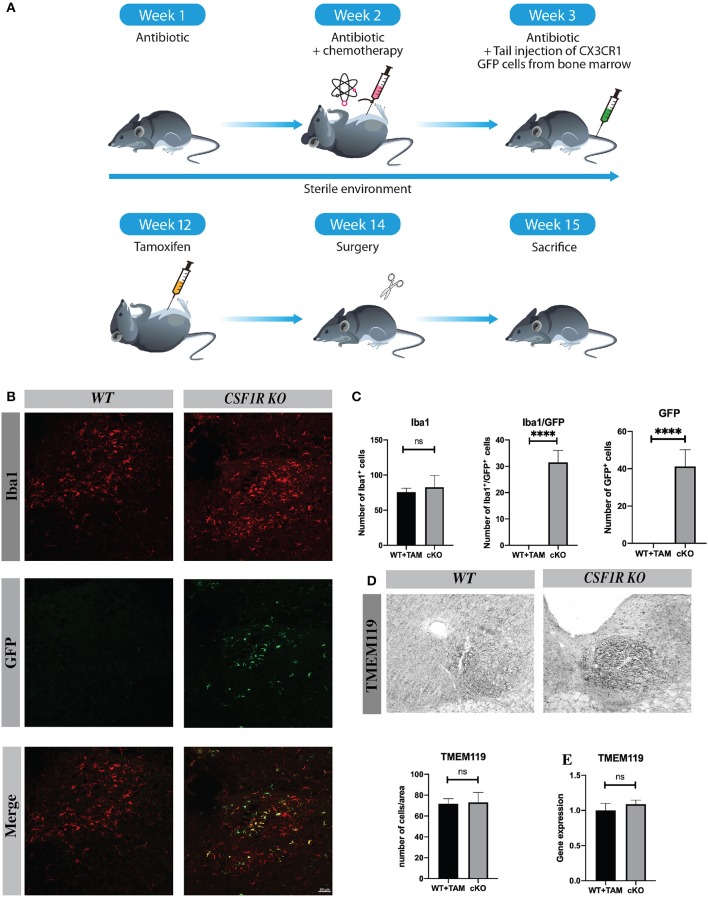
CSF1R depletion induces infiltration of peripheral cells. **(A)** Scheme of the protocol to initiate chimeric mice. **(B)** Images of hypoglossal nucleus showing, infiltrating cells (green) and Iba1 positive cells (red), and infiltrating cells/Iba1 (yellow). Scale bar: **(A)** 50 μm. **(C)** The number of Iba1^+^, GFP^+^, and Iba1^+^/ GFP^+^ was counted in hypoglossal nucleus. **(D)** Tmem119 staining and cells count in hypoglossal nucleus area **(E)** Quantification of Tmem119 gene expression in cerebellum by RT-Q-PCR. Values are expressed as mean ± SEM. Statistical analyses were performed using *t*-test ****p < 0.001 significantly different from cKO group. *n* = 3–5 mice.

These results lead us to wonder if cells from the periphery could compensate and replenish Iba1-positive cells in the ipsilateral hypoglossal nucleus in the CSFR1-deficient mice after TAM injection. We then generated chimeric mice using chemotherapy-based regimen (Figure 4A). This method was used because it does not impact the integrity of the blood-brain barrier, which is limiting the infiltration of bone marrow-derived cells (17). Once the chimerism was confirmed by flow-cytometry, we injected TAM and then 13 days later, mice underwent nerve injury as described in the protocol above. We stained chimeric brains with antibody against Iba-1 to unravel infiltrating GFP-positive cells vs. resident microglia. Unlike wild type animals, numerous GFP cells were found in the brain CSF1R knock-out mice (Figure 4B). Bone marrow-derived cells infiltrated different regions, such as the area postrema and the hypoglossal nucleus. However, the number of Iba1^+^ cells remained similar between wild type and knock-out mice despite the fact that 38.4% of GFP^+^ cells were also Iba-1^+^ in contrast to cuprizone model (Figure 4C). These data are quite interesting because bone marrow-derived cells are naturally attracted when CSF1R is deleted in resident microglia, but the role of these infiltrating cells are not yet well-understood (Figure 4B).

To better understand the role of infiltrating cells we have quantified the number of Tmem119 positive cells in hypoglossal nucleus area and Tmem119 gene expression, this marker is specific to microglia (18) (Figures 4D,E). There was no significative difference in Tmem119 cell count and gene expression between groups suggesting that most proliferating microglia (61.6%) derive from resident cells. Moreover, to complete the study we have verified whether bone marrow-derived cells were positive for Tmem119 and we did not find such positive GFP + Tmem119 cells confirming our findings (Supplementary Figure 1C). We have also determined the expression levels of different chemokines that could be involved in such infiltration process following TAM injection, but we did not see significant changes between WT and cKO brains (data not shown).

### CSF1R-Depleted Microglia Affect Their Proliferation in the Cuprizone Diet Model of Acute Demyelination

**Figure 5:**
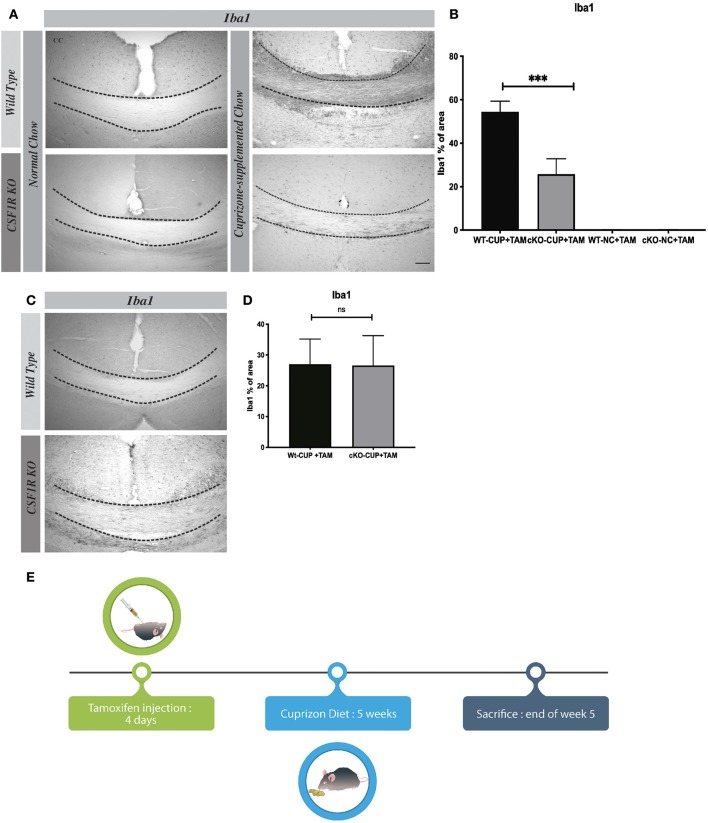
In Cuprizone Diet model CSF1R depleted-microglia affect their proliferation. **(A)** Representative images of Iba1 staining in corpus callosum and **(B)** their quantification presented as percentage of area occupied by the staining, measured in the corpus callosum. Cuprizone supplemented chow (CUP) or Normal Chow (NC). **(C)** Representative images of Iba1 staining in corpus callosum and **(D)** their quantification presented as percentage of area occupied by the staining, measured in the corpus callosum, 5 days after cuprizone withdraw. **(E)** Timeline cuprizone protocol. Value are expressed as means ± SEM. Statistical analyses were performed using *t*-test ***p < 0.001 significantly different from group cKO-CUP + TAM. *N* = 3–6 mice. Scale bar: **(A)** 100 μm.

Cuprizone-induced demyelination is an experimental mouse model to study different pathological events, especially in the progressive forms of MS. Myelin debris induce microglial activation and microgliosis via CSF1R, which plays a critical role in the clearance of myelin debris for a proper remyelination (6). Following a cuprizone diet for 5 weeks, we have deleted CSF1R selectively in microglia using CSF1R^fl/fl^ CX3CR1-Cre^ERT2^ mice. When exposed to tamoxifen, these mice exhibited a significant reduced microgliosis in corpus callosum (Figures 5A,E). The quantification of Iba1^+^ cells shows a significant difference between WT and CSF1R^fl/fl^ CX3CR1-Cre^ERT2^ groups of mice (Figure 5B). It is important to note that the number of Iba-1^+^ cells is no longer different between both groups after the remyelination process several weeks when cuprizone is removed from the diet indicating that CSF1R does not affect survival but proliferation of microglia (Figures 5C,D). These data underline a critical role of CSF1R for microgliosis in this mouse model of progressive MS.

## Discussion

Our results show that inhibition of the CSF1R pathway in microglia in a non-pathologic context does not impair microglial proliferation, which indicates that mCSF receptor is not necessarily implicated in proliferation in this model. CSF1R is known to play a major role in the proliferation and survival of several cell types in various models of diseases (19, 20). Curprizone model mimics the myelin loss observed in MS, especially the primary and secondary progressing form where proliferating microglia play a key role to remove myelin debris to allow a proper remyelination process when mice are no longer exposed to the toxin. In a previous study, CSF1R cKO animals exhibited a heavy myelin debris burden in the corpus callosum along with a reduced number of microglia when compared to their controls. The immune response associated with the remyelination process was also impaired (6).

On the other hand, the hypoglossal nerve lesion is a model to study the marked microglial proliferation in a non-brain pathological context after sectioning the nerve at the peripheral level. Our study aimed to study the role CSF1R pathway in such a phenomenon by deleting the gene using the conditional Cre/Lox system, which was highly efficient and effective as revealed with the CSF1R-loxP-CX3CR1-cre/ERT2, Rosa^tm14^ mice. However, our approaches failed to validate the potential role of the mCSF-CSFR1 pathway in microglial proliferation or survival following several time courses post hypoglossal nerve lesion. It suggests that CSF1R is not involved or is not the only receptor involved in these processes. This may not be explained by the low expression level of the Cre/Lox recombinase since most of Iba1-positive cells in hypoglossal nucleus were also red in the Rosa mice. These data suggest another mechanism underlying the survival and proliferation of resident microglia following a non-pathological brain condition.

Other studies have demonstrated a critical role of CSF1R in microglia survival by using a specific tyrosine kinase inhibitor in healthy mice (21). Using a molecule to shut down a signaling pathway is quite different from using a cell specific inducible gene deletion. The molecule may affect various populations of cells and not specifically microglia, which may explain these different outcomes. Here the Cre/Lox system is restricted to CX3CR1 cells that does not affect their proliferation after hypoglossal nerve lesion but seems to change their phenotypes. Indeed, the structure of CSF1R knock-out microglia are different with their cell bodies that are sharper and darker, and they exhibit more ramifications. Triggering receptor expressed on myeloid cells 2 (TREM2) pathway could be involved in these structural changes, which have been reported in presence of TREM2 overexpression (22). This receptor is also known to take part in microglia activation and survival (16) and there is a link between CSF1R and TREM2 with DAP12. DAP12 is a mediator of CSF1R proliferation pathway through MAPK and Akt. DAP12 ITAM domain is phosphorylated following CSF1R activation triggering ß catenin, a molecule that acts on cellular cycle (1). TREM2/DAP12- mediated signal also promotes proliferation of microglia (22). In this regard, we have observed a significant increased expression of TREM2 in the brain of CSF1R-deficient mice (Figure 3D). The ablation of CSF1R could lead to a compensation by the overexpression of TREM2 and DAP12 phosphorylation in microglia explaining their phenotypic changes without affecting their proliferation.

Interestingly, CSF1R suppression leads to infiltration of circulating CX3CR1-positive cells after the surgery, although most of the proliferating microglia were resident Iba1 cells in hypoglossal nucleus. This was confirmed by the count and the expression levels of transmembrane protein 119 (Tmem 119) mRNA expression, a specific marker of microglia (18) that remained the same between WT and cKO groups (Figures 4D,E). The presence of bone marrow-derived cells is not yet well-understood, as well as the method of recruitment. The chemotherapy-based regiment used to make chimeric mice does not affect the blood-brain barrier (17) and there is no such recruitment in wild-type mice after hypoglossal nerve lesion at any of the time evaluated.

Our study aimed to understand the role of CSF1R in microglial proliferation and survival in a non-pathologic context. We have used two different models, one mimicking the demyelination in progressive MS and in another one of pure and sterile proliferative microglia not associated with pathological conditions in the brain parenchyma. On one hand CSF1R-deleted mice feed with cuprizone exhibited an important drop of microglial cells in corpus callosum area, indicating the vital role of CSF1R signaling pathway for microglia proliferation in this context. On the other hand, CSF1R knock-out mice exhibited a marked microgliosis with no sign of impairment in response to a systemic nerve section. These data are quite novel since most studies that provided solid evidence for the essential role in the mCSF-CSF1R pathway in microgliosis were actually during various pathologies of the CNS. The mechanisms mediating these effects after hypoglossal nerve lesion have yet to be unraveled and whether TREM2 overexpression compensates for CSF1R deletion in microglia will be investigated in future studies.

## Data Availability Statement

The datasets generated for this study are available on request to the corresponding author.

## Ethics Statement

The animal study was reviewed and approved by CPAUL3, Laval University.

## Author Contributions

VP participated in the design of the experiments, analyzed and interpreted the data, wrote the manuscript, and assembled the figures. NL and PP participated in the design of the experiments, analyzed and interpreted the data, and assembled the figures. SR formulated the study concept and all experimental designs, supervised the project, and wrote and revised the manuscript.

### Conflict of Interest

The authors declare that the research was conducted in the absence of any commercial or financial relationships that could be construed as a potential conflict of interest.

## Supplementary Material

Supplementary Figure 1 Timelines. **(A)** Different timelines used for the hypoglossal nerve transection study. **(B)** Timeline used for the cuprizone model. **(C)** Image of hypoglossal nucleus (left) and parenchyma (right) in chimeric mice showing infiltrating cells (green) and Tmem119 (red). Scale bar: 100 μm.
