# Supplementary figures and images for: Role of Macrophage Colony-Stimulating Factor Receptor on the Proliferation and Survival of Microglia Following Systemic Nerve and Cuprizone-Induced Injuries
Source: Front Immunol. 2020 Jan 29;11:47. doi: 10.3389/fimmu.2020.00047 (PMC7001158; doi:10.3389/fimmu.2020.00047)

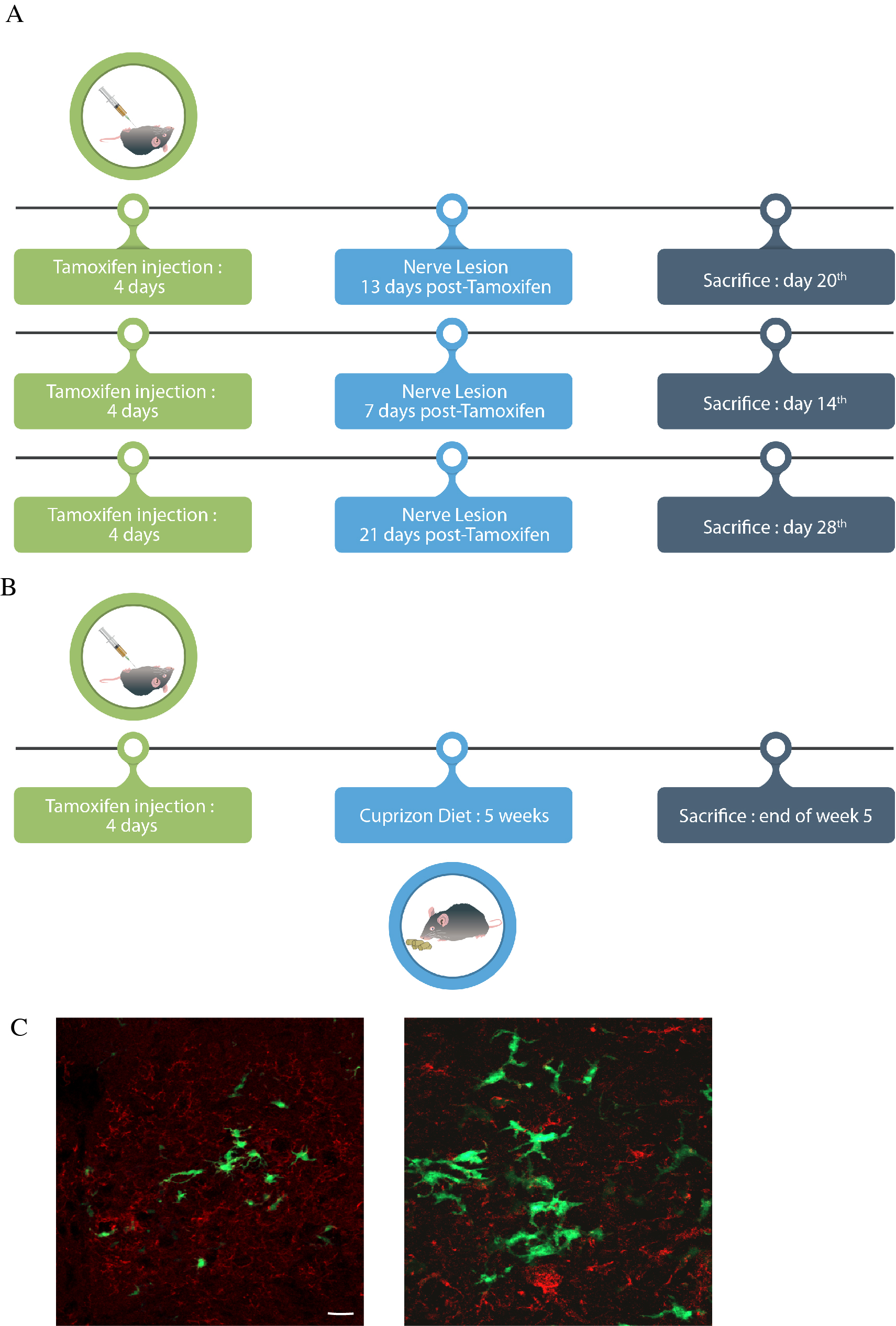

Supplement: Supplementary Figure 1 — Timelines. (A) Different timelines used for the hypoglossal nerve transection study. (B) Timeline used for the cuprizone model. (C) Image of hypoglossal nucleus (left) and parenchyma (right) in chimeric mice showing infiltrating cells (green) and Tmem119 (red). Scale bar: 100 μm. [file Image_1.jpeg]
